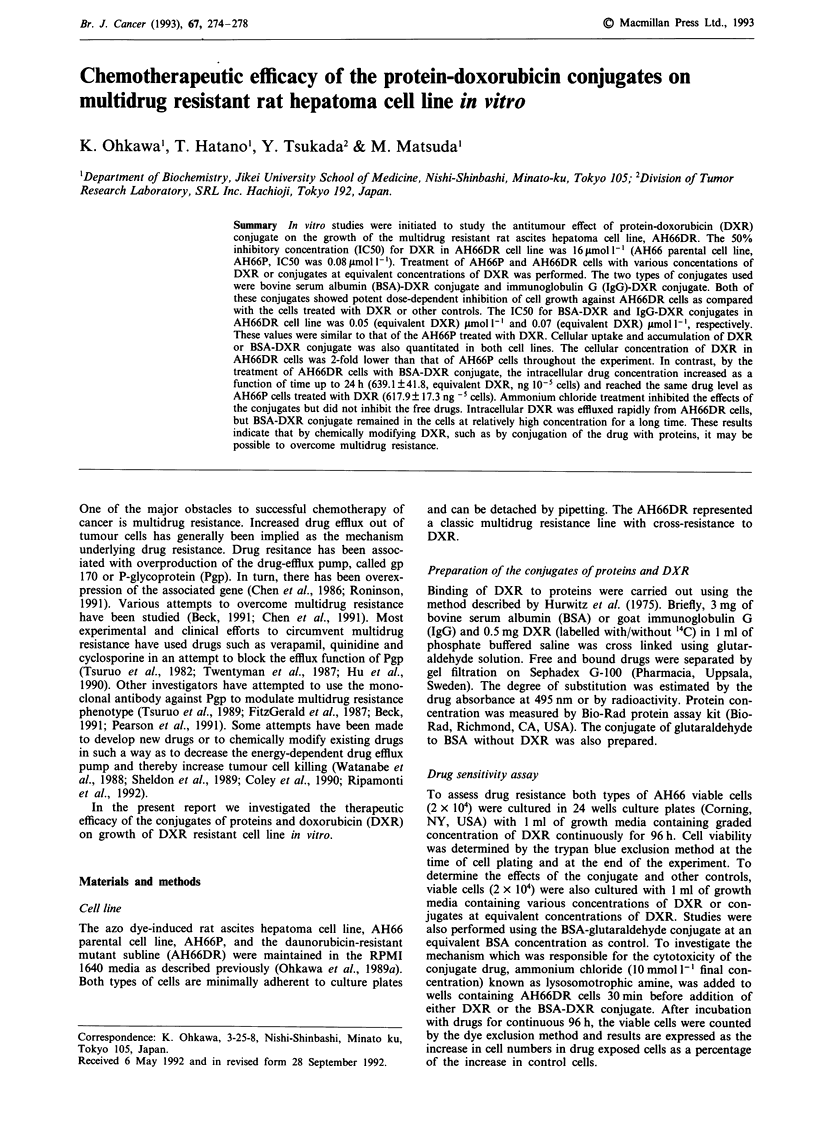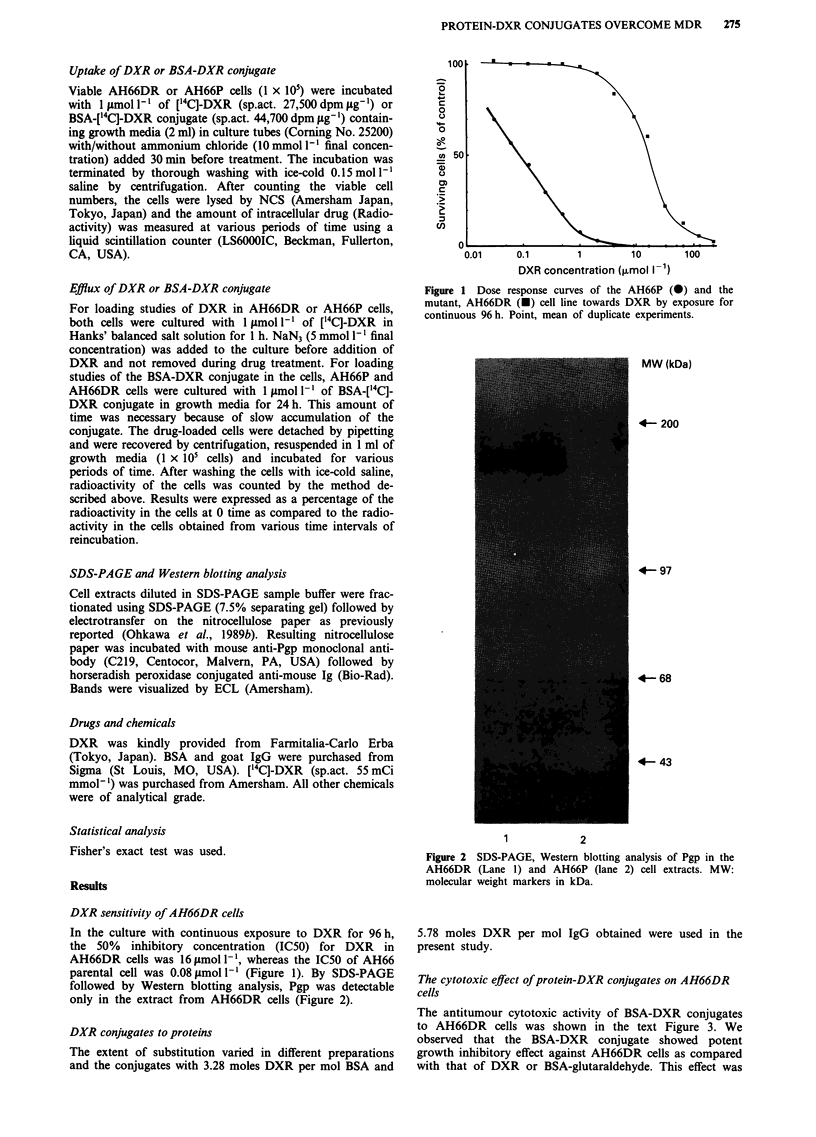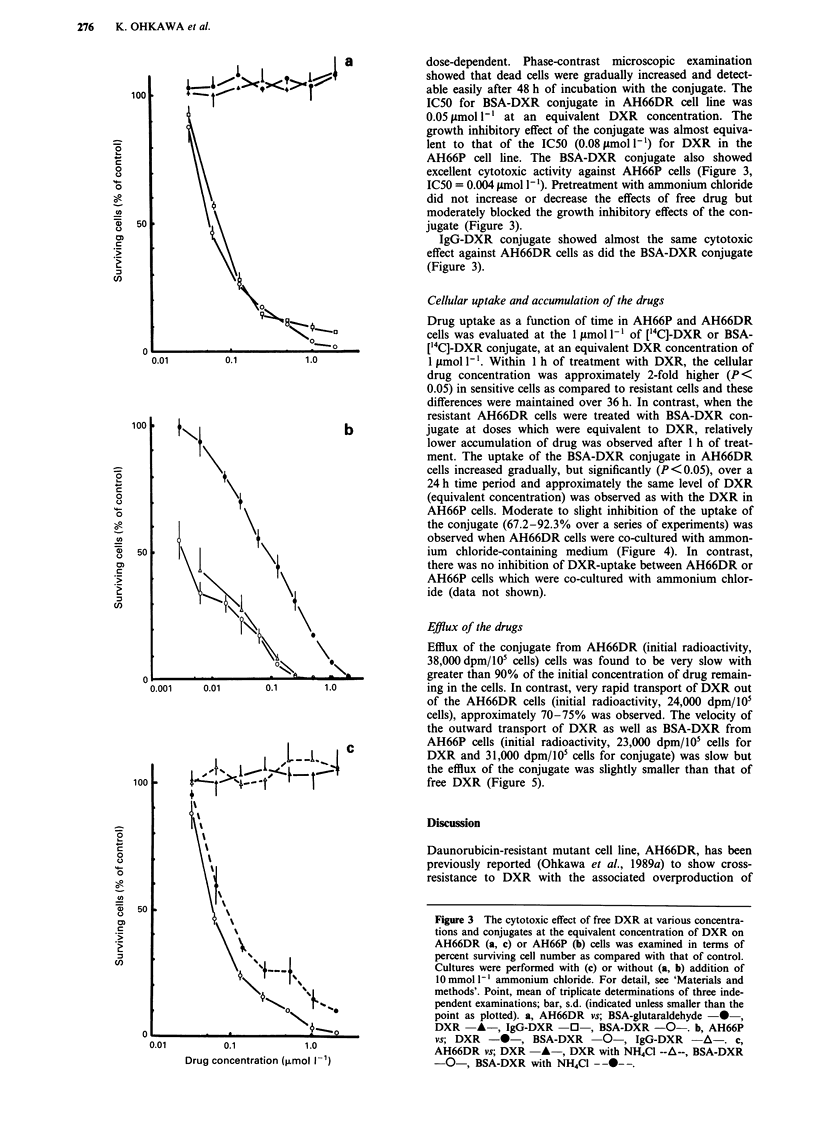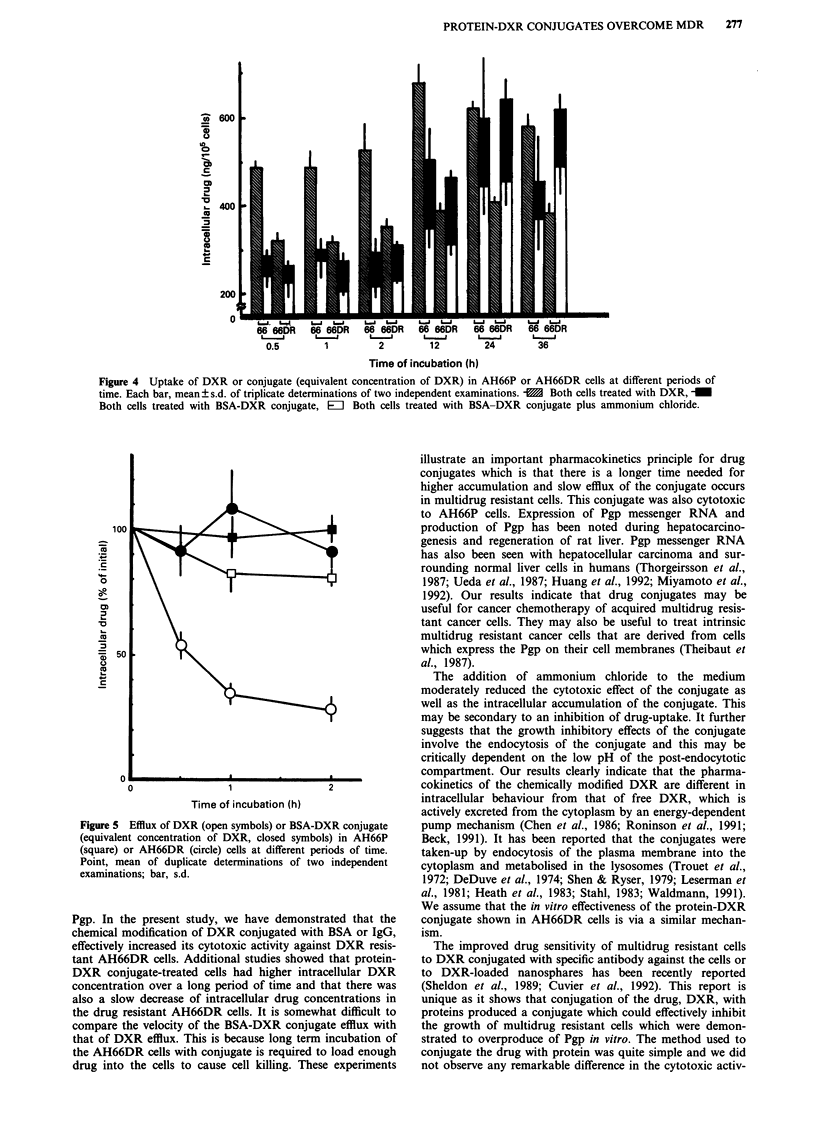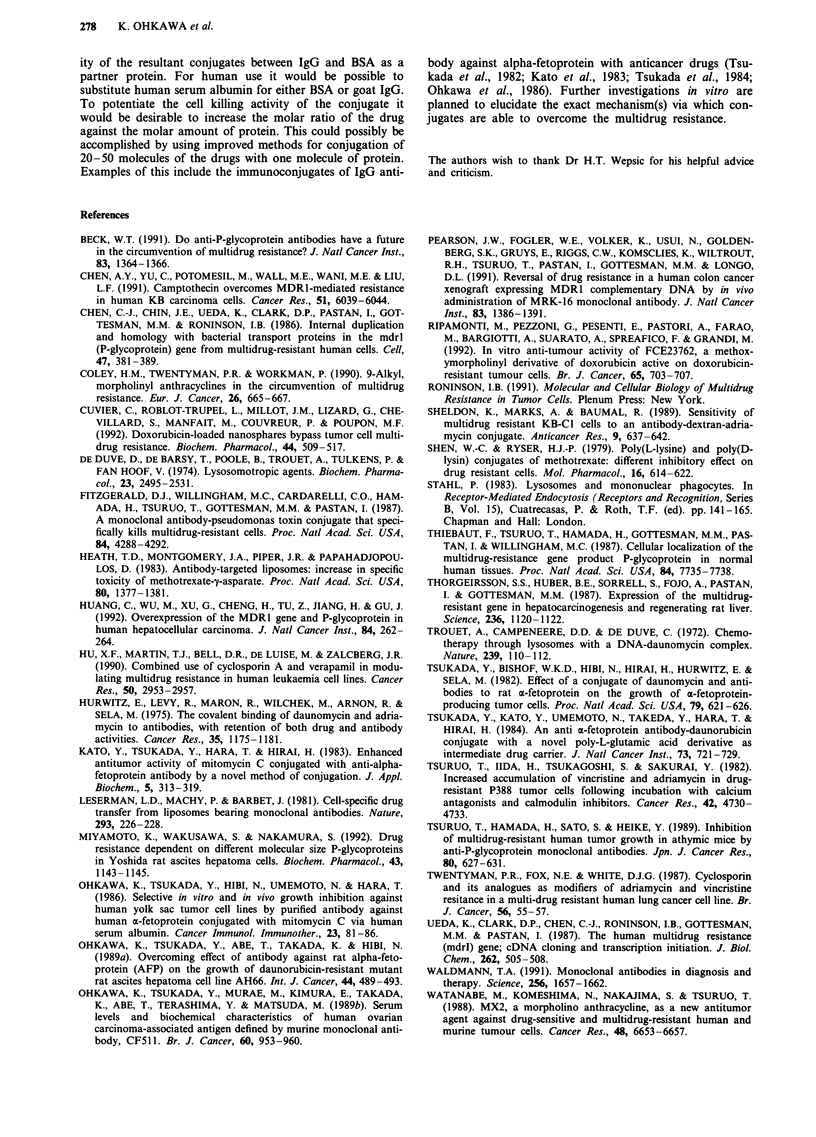# Chemotherapeutic efficacy of the protein-doxorubicin conjugates on multidrug resistant rat hepatoma cell line in vitro.

**DOI:** 10.1038/bjc.1993.52

**Published:** 1993-02

**Authors:** K. Ohkawa, T. Hatano, Y. Tsukada, M. Matsuda

**Affiliations:** Department of Biochemistry, Jikei University School of Medicine, Tokyo, Japan.

## Abstract

**Images:**


Br. J. Cancer (1993), 67, 274–278 © Macmillan Press Ltd., 1993

### Chemotherapeutic efficacy of the protein-doxorubicin conjugates on multidrug resistant rat hepatoma cell line *in vitro*

K. Ohkawa[1], T. Hatano[1], Y. Tsukada[2] & M. Matsuda[1]

[1]*Department of Biochemistry, Jikei University School of Medicine, Nishi-Shinbashi, Minato-ku, Tokyo 105;* [2]*Division of Tumor Research Laboratory, SRL Inc. Hachioji, Tokyo 192, Japan.*

**Summary** *In vitro* studies were initiated to study the antitumour effect of protein-doxorubicin (DXR) conjugate on the growth of the multidrug resistant rat ascites hepatoma cell line, AH66DR. The 50% inhibitory concentration (IC50) for DXR in AH66DR cell line was 16 $\mu mol\ l^{-1}$ (AH66 parental cell line, AH66P, IC50 was 0.08 $\mu mol\ l^{-1}$). Treatment of AH66P and AH66DR cells with various concentations of DXR or conjugates at equivalent concentrations of DXR was performed. The two types of conjugates used were bovine serum albumin (BSA)-DXR conjugate and immunoglobulin G (IgG)-DXR conjugate. Both of these conjugates showed potent dose-dependent inhibition of cell growth against AH66DR cells as compared with the cells treated with DXR or other controls. The IC50 for BSA-DXR and IgG-DXR conjugates in AH66DR cell line was 0.05 (equivalent DXR) $\mu mol\ l^{-1}$ and 0.07 (equivalent DXR) $\mu mol\ l^{-1}$, respectively. These values were similar to that of the AH66P treated with DXR. Cellular uptake and accumulation of DXR or BSA-DXR conjugate was also quantitated in both cell lines. The cellular concentration of DXR in AH66DR cells was 2-fold lower than that of AH66P cells throughout the experiment. In contrast, by the treatment of AH66DR cells with BSA-DXR conjugate, the intracellular drug concentration increased as a function of time up to 24 h (639.1 ± 41.8, equivalent DXR, ng $10^{-5}$ cells) and reached the same drug level as AH66P cells treated with DXR (617.9 ± 17.3 ng $^{-5}$ cells). Ammonium chloride treatment inhibited the effects of the conjugates but did not inhibit the free drugs. Intracellular DXR was effluxed rapidly from AH66DR cells, but BSA-DXR conjugate remained in the cells at relatively high concentration for a long time. These results indicate that by chemically modifying DXR, such as by conjugation of the drug with proteins, it may be possible to overcome multidrug resistance.

One of the major obstacles to successful chemotherapy of cancer is multidrug resistance. Increased drug efflux out of tumour cells has generally been implied as the mechanism underlying drug resistance. Drug resitance has been associated with overproduction of the drug-efflux pump, called gp 170 or P-glycoprotein (Pgp). In turn, there has been overexpression of the associated gene (Chen *et al.*, 1986; Roninson, 1991). Various attempts to overcome multidrug resistance have been studied (Beck, 1991; Chen *et al.*, 1991). Most experimental and clinical efforts to circumvent multidrug resistance have used drugs such as verapamil, quinidine and cyclosporine in an attempt to block the efflux function of Pgp (Tsuruo *et al.*, 1982; Twentyman *et al.*, 1987; Hu *et al.*, 1990). Other investigators have attempted to use the monoclonal antibody against Pgp to modulate multidrug resistance phenotype (Tsuruo *et al.*, 1989; FitzGerald *et al.*, 1987; Beck, 1991; Pearson *et al.*, 1991). Some attempts have been made to develop new drugs or to chemically modify existing drugs in such a way as to decrease the energy-dependent drug efflux pump and thereby increase tumour cell killing (Watanabe *et al.*, 1988; Sheldon *et al.*, 1989; Coley *et al.*, 1990; Ripamonti *et al.*, 1992).

In the present report we investigated the therapeutic efficacy of the conjugates of proteins and doxorubicin (DXR) on growth of DXR resistant cell line *in vitro*.

### Materials and methods

### *Cell line*

The azo dye-induced rat ascites hepatoma cell line, AH66 parental cell line, AH66P, and the daunorubicin-resistant mutant subline (AH66DR) were maintained in the RPMI 1640 media as described previously (Ohkawa *et al.*, 1989*a*). Both types of cells are minimally adherent to culture plates and can be detached by pipetting. The AH66DR represented a classic multidrug resistance line with cross-resistance to DXR.

### *Preparation of the conjugates of proteins and DXR*

Binding of DXR to proteins were carried out using the method described by Hurwitz *et al.* (1975). Briefly, 3 mg of bovine serum albumin (BSA) or goat immunoglobulin G (IgG) and 0.5 mg DXR (labelled with/without $^{14}C$) in 1 ml of phosphate buffered saline was cross linked using glutaraldehyde solution. Free and bound drugs were separated by gel filtration on Sephadex G-100 (Pharmacia, Uppsala, Sweden). The degree of substitution was estimated by the drug absorbance at 495 nm or by radioactivity. Protein concentration was measured by Bio-Rad protein assay kit (Bio-Rad, Richmond, CA, USA). The conjugate of glutaraldehyde to BSA without DXR was also prepared.

### *Drug sensitivity assay*

To assess drug resistance both types of AH66 viable cells ($2 \times 10^4$) were cultured in 24 wells culture plates (Corning, NY, USA) with 1 ml of growth media containing graded concentration of DXR continuously for 96 h. Cell viability was determined by the trypan blue exclusion method at the time of cell plating and at the end of the experiment. To determine the effects of the conjugate and other controls, viable cells ($2 \times 10^4$) were also cultured with 1 ml of growth media containing various concentrations of DXR or conjugates at equivalent concentrations of DXR. Studies were also performed using the BSA-glutaraldehyde conjugate at an equivalent BSA concentration as control. To investigate the mechanism which was responsible for the cytotoxicity of the conjugate drug, ammonium chloride (10 $mmol\ l^{-1}$ final concentration) known as lysosomotrophic amine, was added to wells containing AH66DR cells 30 min before addition of either DXR or the BSA-DXR conjugate. After incubation with drugs for continuous 96 h, the viable cells were counted by the dye exclusion method and results are expressed as the increase in cell numbers in drug exposed cells as a percentage of the increase in control cells.

Correspondence: K. Ohkawa, 3-25-8, Nishi-Shinbashi, Minato ku, Tokyo 105, Japan.
Received 6 May 1992 and in revised form 28 September 1992.

*Uptake of DXR or BSA-DXR conjugate*

Viable AH66DR or AH66P cells ($1 \times 10^5$) were incubated with $1\ \mu mol\ l^{-1}$ of [$^{14}$C]-DXR (sp.act. 27,500 dpm $\mu g^{-1}$) or BSA-[$^{14}$C]-DXR conjugate (sp.act. 44,700 dpm $\mu g^{-1}$) containing growth media (2 ml) in culture tubes (Corning No. 25200) with/without ammonium chloride ($10\ mmol\ l^{-1}$ final concentration) added 30 min before treatment. The incubation was terminated by thorough washing with ice-cold $0.15\ mol\ l^{-1}$ saline by centrifugation. After counting the viable cell numbers, the cells were lysed by NCS (Amersham Japan, Tokyo, Japan) and the amount of intracellular drug (Radioactivity) was measured at various periods of time using a liquid scintillation counter (LS6000IC, Beckman, Fullerton, CA, USA).

*Efflux of DXR or BSA-DXR conjugate*

For loading studies of DXR in AH66DR or AH66P cells, both cells were cultured with $1\ \mu mol\ l^{-1}$ of [$^{14}$C]-DXR in Hanks' balanced salt solution for 1 h. $NaN_3$ ($5\ mmol\ l^{-1}$ final concentration) was added to the culture before addition of DXR and not removed during drug treatment. For loading studies of the BSA-DXR conjugate in the cells, AH66P and AH66DR cells were cultured with $1\ \mu mol\ l^{-1}$ of BSA-[$^{14}$C]-DXR conjugate in growth media for 24 h. This amount of time was necessary because of slow accumulation of the conjugate. The drug-loaded cells were detached by pipetting and were recovered by centrifugation, resuspended in 1 ml of growth media ($1 \times 10^5$ cells) and incubated for various periods of time. After washing the cells with ice-cold saline, radioactivity of the cells was counted by the method described above. Results were expressed as a percentage of the radioactivity in the cells at 0 time as compared to the radioactivity in the cells obtained from various time intervals of reincubation.

*SDS-PAGE and Western blotting analysis*

Cell extracts diluted in SDS-PAGE sample buffer were fractionated using SDS-PAGE (7.5% separating gel) followed by electrotransfer on the nitrocellulose paper as previously reported (Ohkawa *et al.*, 1989*b*). Resulting nitrocellulose paper was incubated with mouse anti-Pgp monoclonal antibody (C219, Centocor, Malvern, PA, USA) followed by horseradish peroxidase conjugated anti-mouse Ig (Bio-Rad). Bands were visualized by ECL (Amersham).

*Drugs and chemicals*

DXR was kindly provided from Farmitalia-Carlo Erba (Tokyo, Japan). BSA and goat IgG were purchased from Sigma (St Louis, MO, USA). [$^{14}$C]-DXR (sp.act. 55 mCi $mmol^{-1}$) was purchased from Amersham. All other chemicals were of analytical grade.

*Statistical analysis*

Fisher's exact test was used.

### Results

*DXR sensitivity of AH66DR cells*

In the culture with continuous exposure to DXR for 96 h, the 50% inhibitory concentration (IC50) for DXR in AH66DR cells was $16\ \mu mol\ l^{-1}$, whereas the IC50 of AH66 parental cell was $0.08\ \mu mol\ l^{-1}$ (Figure 1). By SDS-PAGE followed by Western blotting analysis, Pgp was detectable only in the extract from AH66DR cells (Figure 2).

*DXR conjugates to proteins*

The extent of substitution varied in different preparations and the conjugates with 3.28 moles DXR per mol BSA and 5.78 moles DXR per mol IgG obtained were used in the present study.

**Figure 1** Dose response curves of the AH66P (●) and the mutant, AH66DR (■) cell line towards DXR by exposure for continuous 96 h. Point, mean of duplicate experiments.

**Figure 2** SDS-PAGE, Western blotting analysis of Pgp in the AH66DR (Lane 1) and AH66P (lane 2) cell extracts. MW: molecular weight markers in kDa.

*The cytotoxic effect of protein-DXR conjugates on AH66DR cells*

The antitumour cytotoxic activity of BSA-DXR conjugates to AH66DR cells was shown in the text Figure 3. We observed that the BSA-DXR conjugate showed potent growth inhibitory effect against AH66DR cells as compared with that of DXR or BSA-glutaraldehyde. This effect was dose-dependent. Phase-contrast microscopic examination showed that dead cells were gradually increased and detectable easily after 48 h of incubation with the conjugate. The IC50 for BSA-DXR conjugate in AH66DR cell line was 0.05 μmol l$^{-1}$ at an equivalent DXR concentration. The growth inhibitory effect of the conjugate was almost equivalent to that of the IC50 (0.08 μmol l$^{-1}$) for DXR in the AH66P cell line. The BSA-DXR conjugate also showed excellent cytotoxic activity against AH66P cells (Figure 3, IC50 = 0.004 μmol l$^{-1}$). Pretreatment with ammonium chloride did not increase or decrease the effects of free drug but moderately blocked the growth inhibitory effects of the conjugate (Figure 3).

IgG-DXR conjugate showed almost the same cytotoxic effect against AH66DR cells as did the BSA-DXR conjugate (Figure 3).

*Cellular uptake and accumulation of the drugs*

Drug uptake as a function of time in AH66P and AH66DR cells was evaluated at the 1 μmol l$^{-1}$ of [$^{14}$C]-DXR or BSA-[$^{14}$C]-DXR conjugate, at an equivalent DXR concentration of 1 μmol l$^{-1}$. Within 1 h of treatment with DXR, the cellular drug concentration was approximately 2-fold higher ($P<$ 0.05) in sensitive cells as compared to resistant cells and these differences were maintained over 36 h. In contrast, when the resistant AH66DR cells were treated with BSA-DXR conjugate at doses which were equivalent to DXR, relatively lower accumulation of drug was observed after 1 h of treatment. The uptake of the BSA-DXR conjugate in AH66DR cells increased gradually, but significantly ($P<0.05$), over a 24 h time period and approximately the same level of DXR (equivalent concentration) was observed as with the DXR in AH66P cells. Moderate to slight inhibition of the uptake of the conjugate (67.2–92.3% over a series of experiments) was observed when AH66DR cells were co-cultured with ammonium chloride-containing medium (Figure 4). In contrast, there was no inhibition of DXR-uptake between AH66DR or AH66P cells which were co-cultured with ammonium chloride (data not shown).

*Efflux of the drugs*

Efflux of the conjugate from AH66DR (initial radioactivity, 38,000 dpm/10$^5$ cells) cells was found to be very slow with greater than 90% of the initial concentration of drug remaining in the cells. In contrast, very rapid transport of DXR out of the AH66DR cells (initial radioactivity, 24,000 dpm/10$^5$ cells), approximately 70–75% was observed. The velocity of the outward transport of DXR as well as BSA-DXR from AH66P cells (initial radioactivity, 23,000 dpm/10$^5$ cells for DXR and 31,000 dpm/10$^5$ cells for conjugate) was slow but the efflux of the conjugate was slightly smaller than that of free DXR (Figure 5).

### Discussion

Daunorubicin-resistant mutant cell line, AH66DR, has been previously reported (Ohkawa *et al.*, 1989*a*) to show cross-resistance to DXR with the associated overproduction of

**Figure 3** The cytotoxic effect of free DXR at various concentrations and conjugates at the equivalent concentration of DXR on AH66DR (**a**, **c**) or AH66P (**b**) cells was examined in terms of percent surviving cell number as compared with that of control. Cultures were performed with (**c**) or without (**a**, **b**) addition of 10 mmol l$^{-1}$ ammonium chloride. For detail, see 'Materials and methods'. Point, mean of triplicate determinations of three independent examinations; bar, s.d. (indicated unless smaller than the point as plotted). **a**, AH66DR *vs*; BSA-glutaraldehyde —●—, DXR —▲—, IgG-DXR —□—, BSA-DXR —○—. **b**, AH66P *vs*; DXR —●—, BSA-DXR —○—, IgG-DXR —△—. **c**, AH66DR *vs*; DXR —▲—, DXR with $NH_4Cl$ --△--, BSA-DXR —○—, BSA-DXR with $NH_4Cl$ --●--.

**Figure 4** Uptake of DXR or conjugate (equivalent concentration of DXR) in AH66P or AH66DR cells at different periods of time. Each bar, mean ± s.d. of triplicate determinations of two independent examinations. Both cells treated with DXR, Both cells treated with BSA-DXR conjugate, Both cells treated with BSA–DXR conjugate plus ammonium chloride.

**Figure 5** Efflux of DXR (open symbols) or BSA-DXR conjugate (equivalent concentration of DXR, closed symbols) in AH66P (square) or AH66DR (circle) cells at different periods of time. Point, mean of duplicate determinations of two independent examinations; bar, s.d.

Pgp. In the present study, we have demonstrated that the chemical modification of DXR conjugated with BSA or IgG, effectively increased its cytotoxic activity against DXR resistant AH66DR cells. Additional studies showed that protein-DXR conjugate-treated cells had higher intracellular DXR concentration over a long period of time and that there was also a slow decrease of intracellular drug concentrations in the drug resistant AH66DR cells. It is somewhat difficult to compare the velocity of the BSA-DXR conjugate efflux with that of DXR efflux. This is because long term incubation of the AH66DR cells with conjugate is required to load enough drug into the cells to cause cell killing. These experiments illustrate an important pharmacokinetics principle for drug conjugates which is that there is a longer time needed for higher accumulation and slow efflux of the conjugate occurs in multidrug resistant cells. This conjugate was also cytotoxic to AH66P cells. Expression of Pgp messenger RNA and production of Pgp has been noted during hepatocarcinogenesis and regeneration of rat liver. Pgp messenger RNA has also been seen with hepatocellular carcinoma and surrounding normal liver cells in humans (Thorgeirsson *et al.*, 1987; Ueda *et al.*, 1987; Huang *et al.*, 1992; Miyamoto *et al.*, 1992). Our results indicate that drug conjugates may be useful for cancer chemotherapy of acquired multidrug resistant cancer cells. They may also be useful to treat intrinsic multidrug resistant cancer cells that are derived from cells which express the Pgp on their cell membranes (Theibaut *et al.*, 1987).

The addition of ammonium chloride to the medium moderately reduced the cytotoxic effect of the conjugate as well as the intracellular accumulation of the conjugate. This may be secondary to an inhibition of drug-uptake. It further suggests that the growth inhibitory effects of the conjugate involve the endocytosis of the conjugate and this may be critically dependent on the low pH of the post-endocytotic compartment. Our results clearly indicate that the pharmacokinetics of the chemically modified DXR are different in intracellular behaviour from that of free DXR, which is actively excreted from the cytoplasm by an energy-dependent pump mechanism (Chen *et al.*, 1986; Roninson *et al.*, 1991; Beck, 1991). It has been reported that the conjugates were taken-up by endocytosis of the plasma membrane into the cytoplasm and metabolised in the lysosomes (Trouet *et al.*, 1972; DeDuve *et al.*, 1974; Shen & Ryser, 1979; Leserman *et al.*, 1981; Heath *et al.*, 1983; Stahl, 1983; Waldmann, 1991). We assume that the *in vitro* effectiveness of the protein-DXR conjugate shown in AH66DR cells is via a similar mechanism.

The improved drug sensitivity of multidrug resistant cells to DXR conjugated with specific antibody against the cells or to DXR-loaded nanospheres has been recently reported (Sheldon *et al.*, 1989; Cuvier *et al.*, 1992). This report is unique as it shows that conjugation of the drug, DXR, with proteins produced a conjugate which could effectively inhibit the growth of multidrug resistant cells which were demonstrated to overproduce of Pgp *in vitro*. The method used to conjugate the drug with protein was quite simple and we did not observe any remarkable difference in the cytotoxic activity of the resultant conjugates between IgG and BSA as a partner protein. For human use it would be possible to substitute human serum albumin for either BSA or goat IgG. To potentiate the cell killing activity of the conjugate it would be desirable to increase the molar ratio of the drug against the molar amount of protein. This could possibly be accomplished by using improved methods for conjugation of 20–50 molecules of the drugs with one molecule of protein. Examples of this include the immunoconjugates of IgG antibody against alpha-fetoprotein with anticancer drugs (Tsukada *et al.*, 1982; Kato *et al.*, 1983; Tsukada *et al.*, 1984; Ohkawa *et al.*, 1986). Further investigations *in vitro* are planned to elucidate the exact mechanism(s) via which conjugates are able to overcome the multidrug resistance.

The authors wish to thank Dr H.T. Wepsic for his helpful advice and criticism.

### References

BECK, W.T. (1991). Do anti-P-glycoprotein antibodies have a future in the circumvention of multidrug resistance? *J. Natl Cancer Inst.*, **83**, 1364–1366.

CHEN, A.Y., YU, C., POTOMESIL, M., WALL, M.E., WANI, M.E. & LIU, L.F. (1991). Camptothecin overcomes MDR1-mediated resistance in human KB carcinoma cells. *Cancer Res.*, **51**, 6039–6044.

CHEN, C.-J., CHIN, J.E., UEDA, K., CLARK, D.P., PASTAN, I., GOTTESMAN, M.M. & RONINSON, I.B. (1986). Internal duplication and homology with bacterial transport proteins in the mdr1 (P-glycoprotein) gene from multidrug-resistant human cells. *Cell*, **47**, 381–389.

COLEY, H.M., TWENTYMAN, P.R. & WORKMAN, P. (1990). 9-Alkyl, morpholinyl anthracyclines in the circumvention of multidrug resistance. *Eur. J. Cancer*, **26**, 665–667.

CUVIER, C., ROBLOT-TRUPEL, L., MILLOT, J.M., LIZARD, G., CHEVILLARD, S., MANFAIT, M., COUVREUR, P. & POUPON, M.F. (1992). Doxorubicin-loaded nanospheres bypass tumor cell multidrug resistance. *Biochem. Pharmacol.*, **44**, 509–517.

DE DUVE, D., DE BARSY, T., POOLE, B., TROUET, A., TULKENS, P. & FAN HOOF, V. (1974). Lysosomotropic agents. *Biochem. Pharmacol.*, **23**, 2495–2531.

FITZGERALD, D.J., WILLINGHAM, M.C., CARDARELLI, C.O., HAMADA, H., TSURUO, T., GOTTESMAN, M.M. & PASTAN, I. (1987). A monoclonal antibody-pseudomonas toxin conjugate that specifically kills multidrug-resistant cells. *Proc. Natl Acad. Sci. USA*, **84**, 4288–4292.

HEATH, T.D., MONTGOMERY, J.A., PIPER, J.R. & PAPAHADJOPOULOS, D. (1983). Antibody-targeted liposomes: increase in specific toxicity of methotrexate-γ-asparate. *Proc. Natl Acad. Sci. USA*, **80**, 1377–1381.

HUANG, C., WU, M., XU, G., CHENG, H., TU, Z., JIANG, H. & GU, J. (1992). Overexpression of the MDR1 gene and P-glycoprotein in human hepatocellular carcinoma. *J. Natl Cancer Inst.*, **84**, 262–264.

HU, X.F., MARTIN, T.J., BELL, D.R., DE LUISE, M. & ZALCBERG, J.R. (1990). Combined use of cyclosporin A and verapamil in modulating multidrug resistance in human leukaemia cell lines. *Cancer Res.*, **50**, 2953–2957.

HURWITZ, E., LEVY, R., MARON, R., WILCHEK, M., ARNON, R. & SELA, M. (1975). The covalent binding of daunomycin and adriamycin to antibodies, with retention of both drug and antibody activities. *Cancer Res.*, **35**, 1175–1181.

KATO, Y., TSUKADA, Y., HARA, T. & HIRAI, H. (1983). Enhanced antitumor activity of mitomycin C conjugated with anti-alpha-fetoprotein antibody by a novel method of conjugation. *J. Appl. Biochem.*, **5**, 313–319.

LESERMAN, L.D., MACHY, P. & BARBET, J. (1981). Cell-specific drug transfer from liposomes bearing monoclonal antibodies. *Nature*, **293**, 226–228.

MIYAMOTO, K., WAKUSAWA, S. & NAKAMURA, S. (1992). Drug resistance dependent on different molecular size P-glycoproteins in Yoshida rat ascites hepatoma cells. *Biochem. Pharmacol.*, **43**, 1143–1145.

OHKAWA, K., TSUKADA, Y., HIBI, N., UMEMOTO, N. & HARA, T. (1986). Selective *in vitro* and *in vivo* growth inhibition against human yolk sac tumor cell lines by purified antibody against human α-fetoprotein conjugated with mitomycin C via human serum albumin. *Cancer Immunol. Immunother.*, **23**, 81–86.

OHKAWA, K., TSUKADA, Y., ABE, T., TAKADA, K. & HIBI, N. (1989*a*). Overcoming effect of antibody against rat alpha-fetoprotein (AFP) on the growth of daunorubicin-resistant mutant rat ascites hepatoma cell line AH66. *Int. J. Cancer*, **44**, 489–493.

OHKAWA, K., TSUKADA, Y., MURAE, M., KIMURA, E., TAKADA, K., ABE, T., TERASHIMA, Y. & MATSUDA, M. (1989*b*). Serum levels and biochemical characteristics of human ovarian carcinoma-associated antigen defined by murine monoclonal antibody, CF511. *Br. J. Cancer*, **60**, 953–960.

PEARSON, J.W., FOGLER, W.E., VOLKER, K., USUI, N., GOLDENBERG, S.K., GRUYS, E., RIGGS, C.W., KOMSCLIES, K., WILTROUT, R.H., TSURUO, T., PASTAN, I., GOTTESMAN, M.M. & LONGO, D.L. (1991). Reversal of drug resistance in a human colon cancer xenograft expressing MDR1 complementary DNA by *in vivo* administration of MRK-16 monoclonal antibody. *J. Natl Cancer Inst.*, **83**, 1386–1391.

RIPAMONTI, M., PEZZONI, G., PESENTI, E., PASTORI, A., FARAO, M., BARGIOTTI, A., SUARATO, A., SPREAFICO, F. & GRANDI, M. (1992). In vitro anti-tumour activity of FCE23762, a methoxymorpholinyl derivative of doxorubicin active on doxorubicin-resistant tumour cells. *Br. J. Cancer*, **65**, 703–707.

RONINSON, I.B. (1991). *Molecular and Cellular Biology of Multidrug Resistance in Tumor Cells*. Plenum Press: New York.

SHELDON, K., MARKS, A. & BAUMAL, R. (1989). Sensitivity of multidrug resistant KB-C1 cells to an antibody-dextran-adriamycin conjugate. *Anticancer Res.*, **9**, 637–642.

SHEN, W.-C. & RYSER, H.J.-P. (1979). Poly(L-lysine) and poly(D-lysin) conjugates of methotrexate: different inhibitory effect on drug resistant cells. *Mol. Pharmacol.*, **16**, 614–622.

STAHL, P. (1983). Lysosomes and mononuclear phagocytes. In *Receptor-Mediated Endocytosis (Receptors and Recognition*, Series B, Vol. 15), Cuatrecasas, P. & Roth, T.F. (ed). pp. 141–165. Chapman and Hall: London.

THIEBAUT, F., TSURUO, T., HAMADA, H., GOTTESMAN, M.M., PASTAN, I. & WILLINGHAM, M.C. (1987). Cellular localization of the multidrug-resistance gene product P-glycoprotein in normal human tissues. *Proc. Natl Acad. Sci. USA*, **84**, 7735–7738.

THORGEIRSSON, S.S., HUBER, B.E., SORRELL, S., FOJO, A., PASTAN, I. & GOTTESMAN, M.M. (1987). Expression of the multidrug-resistant gene in hepatocarcinogenesis and regenerating rat liver. *Science*, **236**, 1120–1122.

TROUET, A., CAMPENEERE, D.D. & DE DUVE, C. (1972). Chemotherapy through lysosomes with a DNA-daunomycin complex. *Nature*, **239**, 110–112.

TSUKADA, Y., BISHOF, W.K.D., HIBI, N., HIRAI, H., HURWITZ, E. & SELA, M. (1982). Effect of a conjugate of daunomycin and antibodies to rat α-fetoprotein on the growth of α-fetoprotein-producing tumor cells. *Proc. Natl Acad. Sci. USA*, **79**, 621–626.

TSUKADA, Y., KATO, Y., UMEMOTO, N., TAKEDA, Y., HARA, T. & HIRAI, H. (1984). An anti α-fetoprotein antibody-daunorubicin conjugate with a novel poly-L-glutamic acid derivative as intermediate drug carrier. *J. Natl Cancer Inst.*, **73**, 721–729.

TSURUO, T., IIDA, H., TSUKAGOSHI, S. & SAKURAI, Y. (1982). Increased accumulation of vincristine and adriamycin in drug-resistant P388 tumor cells following incubation with calcium antagonists and calmodulin inhibitors. *Cancer Res.*, **42**, 4730–4733.

TSURUO, T., HAMADA, H., SATO, S. & HEIKE, Y. (1989). Inhibition of multidrug-resistant human tumor growth in athymic mice by anti-P-glycoprotein monoclonal antibodies. *Jpn. J. Cancer Res.*, **80**, 627–631.

TWENTYMAN, P.R., FOX, N.E. & WHITE, D.J.G. (1987). Cyclosporin and its analogues as modifiers of adriamycin and vincristine resistance in a multi-drug resistant human lung cancer cell line. *Br. J. Cancer*, **56**, 55–57.

UEDA, K., CLARK, D.P., CHEN, C.-J., RONINSON, I.B., GOTTESMAN, M.M. & PASTAN, I. (1987). The human multidrug resistance (mdrI) gene; cDNA cloning and transcription initiation. *J. Biol. Chem.*, **262**, 505–508.

WALDMANN, T.A. (1991). Monoclonal antibodies in diagnosis and therapy. *Science*, **256**, 1657–1662.

WATANABE, M., KOMESHIMA, N., NAKAJIMA, S. & TSURUO, T. (1988). MX2, a morpholino anthracycline, as a new antitumor agent against drug-sensitive and multidrug-resistant human and murine tumour cells. *Cancer Res.*, **48**, 6653–6657.